# OmpC and OmpF Outer Membrane Proteins of *Escherichia coli* and *Salmonella enterica* Form *Bona Fide* Amyloids

**DOI:** 10.3390/ijms242115522

**Published:** 2023-10-24

**Authors:** Mikhail V. Belousov, Anastasiia O. Kosolapova, Haidar Fayoud, Maksim I. Sulatsky, Anna I. Sulatskaya, Maria N. Romanenko, Alexander G. Bobylev, Kirill S. Antonets, Anton A. Nizhnikov

**Affiliations:** 1 All-Russia Research Institute for Agricultural Microbiology, 196608 St. Petersburg, Russia; m.belousov@arriam.ru (M.V.B.); kosolapova97@mail.ru (A.O.K.); haidar.fayoud@gmail.com (H.F.); m.romanenko@arriam.ru (M.N.R.); k.antonets@arriam.ru (K.S.A.); 2 Faculty of Biology, St. Petersburg State University, 199034 St. Petersburg, Russia; 3 Institute of Cytology, Russian Academy of Sciences, 194064 St. Petersburg, Russia; m_sulatsky@mail.ru (M.I.S.); ansul@mail.ru (A.I.S.); 4 Institute of Theoretical and Experimental Biophysics, Russian Academy of Sciences, 142290 Pushchino, Russia; bobylev1982@gmail.com

**Keywords:** amyloid, fibril, porin, outer membrane proteins, Omp, virulence, host–pathogen, bacteria, *Escherichia coli*, *Salmonella enterica*

## Abstract

Outer membrane proteins (Omps) of Gram-negative bacteria represent porins involved in a wide range of virulence- and pathogenesis-related cellular processes, including transport, adhesion, penetration, and the colonization of host tissues. Most outer membrane porins share a specific spatial structure called the β-barrel that provides their structural integrity within the membrane lipid bilayer. Recent data suggest that outer membrane proteins from several bacterial species are able to adopt the amyloid state alternative to their β-barrel structure. Amyloids are protein fibrils with a specific spatial structure called the cross-β that gives them an unusual resistance to different physicochemical influences. Various bacterial amyloids are known to be involved in host-pathogen and host-symbiont interactions and contribute to colonization of host tissues. Such an ability of outer membrane porins to adopt amyloid state might represent an important mechanism of bacterial virulence. In this work, we investigated the amyloid properties of the OmpC and OmpF porins from two species belonging to *Enterobacteriaceae* family, *Escherichia coli*, and *Salmonella enterica.* We demonstrated that OmpC and OmpF of *E. coli* and *S. enterica* form toxic fibrillar aggregates in vitro. These aggregates exhibit birefringence upon binding Congo Red dye and show characteristic reflections under X-ray diffraction. Thus, we confirmed amyloid properties for OmpC of *E. coli* and demonstrated *bona fide* amyloid properties for three novel proteins: OmpC of *S. enterica* and OmpF of *E. coli* and *S. enterica* in vitro. All four studied porins were shown to form amyloid fibrils at the surface of *E. coli* cells in the curli-dependent amyloid generator system. Moreover, we found that overexpression of recombinant OmpC and OmpF in the *E. coli* BL21 strain leads to the formation of detergent- and protease-resistant amyloid-like aggregates and enhances the birefringence of bacterial cultures stained with Congo Red. We also detected detergent- and protease-resistant aggregates comprising OmpC and OmpF in *S. enterica* culture. These data are important in the context of understanding the structural dualism of Omps and its relation to pathogenesis.

## 1. Introduction

Cells of Gram-negative bacteria of the *Enterobacteriaceae* family, such as *Escherichia coli* and *Salmonella enterica*, are covered by two lipid bilayer membranes that delimit the periplasmic space. Both membranes, but mostly the outer one, contain pore proteins that facilitate the transport of various substances [1]. These membranes differ markedly in composition and function. With regard to function, the main difference is that the outer membrane, due to the presence of pore-forming proteins (porins), is significantly more permeable than the cytoplasmic membrane [2,3]. Outer membrane proteins (hereinafter Omps) are highly conserved [4] and complexly regulated [5]. They are involved in different processes associated with virulence and pathogenesis of enterobacteria: adhesion, penetration, colonization, and damage to host tissues, as well as the avoidance of the host immune response [5,6,7,8]. While common non-specific porins like OmpA, OmpC, and OmpF facilitate the diffusion of hydrophilic molecules (<600 Da) [9] and do not show much substrate specificity despite some selectivity for either cations or anions [10], there is a group of specific porins that are selective for certain substrates [2].

The expression of Omps is controlled by complex regulatory pathways in a genotype- and condition-dependent manner [11]. The synthesis of several porins, such as trimeric OmpC and OmpF, in *E. coli* cells is affected by the osmolarity of the cultural medium in a reciprocal manner [12]. It should be noted that, though deletions of genes encoding OmpC and OmpF are viable in *E. coli*, they significantly decrease the resistance to stressful conditions [13]. It is also worth noting that despite the significant amino acid sequence similarity between these porins in *E. coli* [14], the difference in pore sizes reaches 0.1 nm (1.1 and 1.2 nm for OmpC and OmpF, respectively [15]), which, being seemingly slight, contributes, for example, to a two-fold decrease in the rate of glucose permeability [5]. Also, the conductivity of the OmpC and OmpF channels depends on the pH and salt composition of the medium [16], membrane surface stress [17], temperature [18], etc. The reciprocal expression of *ompC* and *ompF* controlled by the OmpR transcriptional regulator and providing different outer membrane permeability is important for *E. coli* to survive in two different conditions: high osmolarity (within a host gut, where the expression of OmpC with a lower pore size increases) and low osmolarity (in the external environment, where the expression of OmpF with a higher pore size increases) [19]. Additionally, *ompC* and *ompF* exhibit pH-dependent regulation of expression: at an acidic pH, the levels of OmpC increase while those of OmpF decrease [20].

Nearly forty years ago, the OmpF porin became the first membrane protein to obtain crystals of a size and quality that could be subjected to high-resolution structural analysis using X-ray crystallography [21]. However, more than 10 years passed before its atomic structure was elucidated [22]. Currently, OmpC and OmpF are known to possess β-barrel structures in their functionally active porin states [22]. Nevertheless, a few years ago OmpC of *E. coli*, as well as several other porins and porin-like proteins, were shown to form amyloids or amyloid-like aggregates [8,23,24]. Amyloids represent insoluble fibrillar protein aggregates involved in a wide range of pathogenic and functional processes [25,26,27] and possessing a common spatial structure called cross-β, which determines the unique properties of these protein assemblies: a cross-β pattern under X-ray diffraction, apple-green birefringence in polarized light upon staining with Congo Red (CR) dye, a change in photophysical properties of Thioflavin T (ThT) dye, and resistance to proteases and ionic detergents [26]. Thus, several Omps are likely to adopt two different conformational states: β-barrel pore and cross-β fold, resulting in amyloid formation. Though the functional significance of the amyloid formation by Omps remains unclear, such a structural dualism of these proteins represents an interesting phenomenon that needs further investigation and could be related to modulation of bacterial virulence, toxicity, and host-pathogen interactions.

Previously, we identified OmpC and OmpF proteins in the fraction of detergent-resistant protein polymers and complexes isolated from the *E. coli* cells that were resistant to treatment with ionic detergents [28]. The analysis of the amyloid properties of the *E. coli* OmpC performed by another research group demonstrated its amyloid properties in vitro and the toxicity of aggregates to mammals [24]. Here, we continue to study the amyloid properties of the Omps of *Enterobacteriaceae*. We have performed an in-depth study of the amyloid properties of OmpC and OmpF from two enterobacterial species, *E. coli* and *S. enterica subsp. enterica* var. Issatschenko (hereinafter referred as *S. enterica*). We have confirmed the amyloid properties of the OmpC of *E. coli* and demonstrated that the OmpC of *S. enterica*, as well as the OmpF of *E. coli* and *S. enterica*, form *bona fide* amyloid fibrils toxic to mammalian cells.

## 2. Results

### 2.1. OmpC and OmpF of E. coli and S. enterica form Fibrillar Aggregates In Vitro

**Figure 1:**
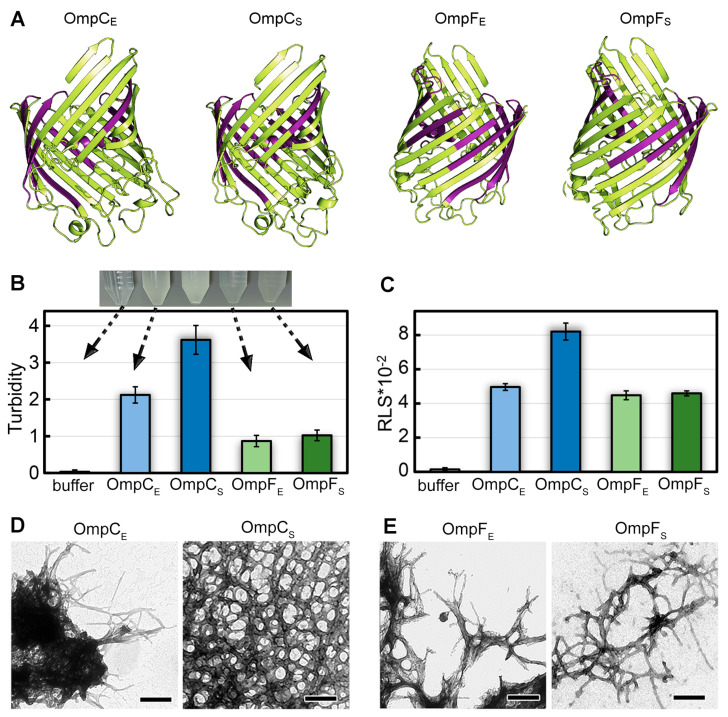
The structures of OmpC and OmpF of *E. coli* and *S. enterica* and morphological features of aggregates formed by these proteins in vitro. (**A**) The structures of OmpC and OmpF from *E. coli* and *S. enterica* predicted by AlphaFold. Designations “E” and “S” in the protein names hereinafter correspond to orthologs from *E. coli* and *S. enterica*, respectively. The potentially amyloidogenic regions predicted by AmylPred2 are denoted in purple. (**B**) Turbidity and (**C**) Rayleigh light scattering (RLS) of Omp aggregates. Parameters for the buffer are given as a control. At the top of (**B**), the buffer solution and Omp samples after fibrillogenesis are visualized. The arrows indicate the turbidity values of each sample. (**D**,**E**) TEM images of the aggregates formed from (**D**) OmpC and (**E**) OmpF obtained from *E. coli* (left panels) and *S. enterica* (right panels). The scale bars are equal to 200 nm.

The OmpC and OmpF proteins of *E. coli* and *S. enterica* share a similar spatial structure containing a large β-barrel domain (Figure 1A); these proteins have significant amino acid sequence identity and similarity [29,30] and similar physicochemical properties (Mw 38.8–40.3 kDa, pI 4.6–4.8) (Table S1). All four proteins, according to prediction by AmyPred2 [31], have large potentially amyloidogenic regions (Table S2) located within the β-barrel domain (Figure 1A). These features of these Omps’ structures make them interesting candidates for a comparative analysis of their amyloid properties.

To analyze the ability of OmpC and OmpF proteins of *E. coli* and *S. enterica* to form fibrillar aggregates in vitro, we produced C-terminally 6×-His-tagged full-length proteins without N-terminal signal peptides in *E. coli* and extracted and purified them. To perform fibrillogenesis in vitro, proteins were first monomerized in 50% 1,1,1,3,3,3-Hexafluoro-2-propanol (HFIP). After the removal of this organic solvent from the sample, the Omps were incubated in Milli-Q water with constant stirring at a temperature of 37 °C. This protocol was successfully used previously to obtain amyloid fibrils from human β-amyloid peptide [32], garden pea *Pisum sativum* L. vicilin, as well as root nodule bacterium *Rhizobium leguminosarum* RopA and RopB proteins [33,34]. After two weeks of incubation, when all the studied samples became opalescent (Figure 1B, top), we analyzed them using a set of spectroscopic and microscopic methods.

The difference in turbidity (Figure 1B) and Rayleigh light scattering (RLS, Figure 1C) values of the samples indicates a difference in the number and/or size of aggregates formed from different Omps. In particular, it turned out that suspensions with OmpC aggregates have noticeably higher values of turbidity than OmpF samples. At the same time, the samples with OmpC of *E. coli* and *S. enterica* are characterized by different values of RLS and turbidity in contrast to the OmpF samples (Figure 1B,C). Transmission electron microscopy was used to visualize the prepared aggregates (Figure 1D,E). Protein fibrils of various thicknesses were found in all samples. The morphology of *S. enterica* OmpC fibrils differed most from other samples as they represented a dense network of intertwined fibrils. This is probably the reason for the highest RLS and turbidity for these fibrils. Other fibrils appear similar in morphology: thin, bendable filaments prone to clustering. The largest clots were formed by OmpC fibrils from *E. coli*, which is in good agreement with the high values of their turbidity and RLS. Detailed micrographs of Omps fibrils at higher magnification were also obtained (Figure S1A–D). A widely used model object, lysozyme amyloid fibrils, was used as a positive control (Figure S1G,H shows that the morphology of lysozyme amyloid fibrils and aggregates of Omps is similar). Soluble lysozyme (Figure S1E) and its amorphous aggregates (Figure S1F) were used as negative controls.

### 2.2. The In Vitro Formed Aggregates of the E. coli and S. enterica OmpC and OmpF Bind CR and ThT Dyes

**Figure 2:**
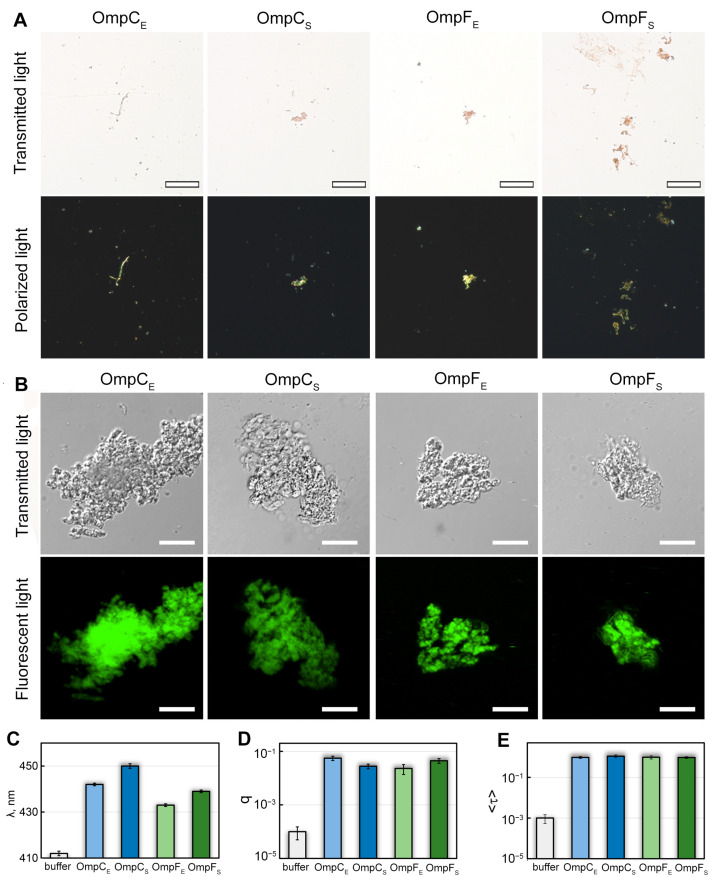
Interaction of OmpC and OmpF aggregates with CR and ThT dyes. (**A**) Polarized light microscopy of the Omp aggregates stained with CR. Top row: transmitted light; bottom: polarized light. The scale bar is equal to 50 μm. (**B**) Confocal microscopy of the Omp aggregates stained with Thioflavin T (ThT). Top row: transmitted light; bottom: fluorescent light. The scale bars are equal to 10 μm. (**C**) Wavelength of the absorption spectrum maximum (λ), (**D**) fluorescence quantum yield (q), and (**E**) fluorescence lifetime (<τ>) of ThT bound to Omp fibrils. Characteristics of ThT in the buffer are given as a control. Designations “E” and “S” in the names of proteins correspond to the orthologs from *E. coli* and *S. enterica*, respectively.

The specific property of amyloids is the apple-green (in several cases, green, yellow, and orange) birefringence occurring in the polarized light upon binding CR dye [35,36]. We stained in-vitro-obtained OmpC and OmpF fibrillar aggregates with CR and analyzed the effects of staining using polarized light microscopy. All four samples were found to bind CR and exhibited a red color in transmitted light (Figure 2A). In the polarized light, we detected green birefringence in all studied samples, suggesting amyloid properties in them (Figure 2A).

We also stained in-vitro-obtained OmpC and OmpF aggregates with ThT dye, whose binding to amyloids changes its photophysical properties [37]. We found the aggregates present in all four samples bound to ThT (Figure 2B). To analyze the specificity of ThT binding to Omp aggregates, we studied dye-aggregate samples prepared by the equilibrium microdialysis approach specially proposed by us for such purposes [38]. It turned out that when ThT binds to Omp fibrils, the absorption spectrum of the dye (Figure 2C) is significantly shifted to the long wavelength region (by tens of nm), and its fluorescence quantum yield (Figure 2D) and the fluorescence lifetime (Figure 2E) increase by 2 and 3 orders of magnitude, respectively, compared with those for free ThT in aqueous solution. According to literature (see, for example, [39,40,41]), the detected changes in the photophysical properties of ThT are characteristic of the dye binding to *bona fide* amyloid fibrils, which indicates the amyloid nature of all tested aggregates. At the same time, ThT absorption spectrum and fluorescence quantum yield differ in the case of various Omp amyloids (Figure 2C,D), which indicates differences in their structure.

### 2.3. The Aggregates of the E. coli and S. enterica OmpC and OmpF Obtained In Vitro Exhibit Resistance to Treatment with Cold Ionic Detergent SDS and Trypsin Protease

**Figure 3:**
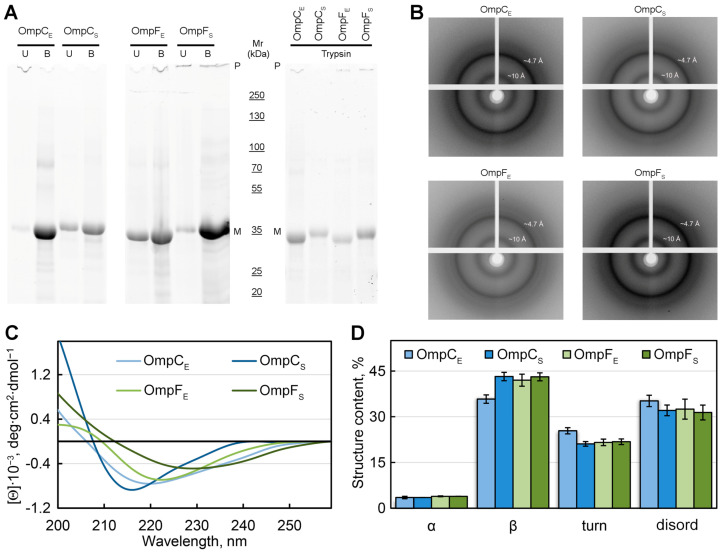
Analysis of the structure of in-vitro-obtained aggregates of Omps and their detergent and protease resistance. (**A**) The resistance of the Omp aggregates to treatment with room temperature (U, unboiled) and hot (B, boiled) 2% SDS and to trypsin treatment (at 1:60 trypsin-to-total protein mass ratio for 20 min at 37 °C). Gel stained using Stain-Free™ technology (Bio-Rad, Hercules, CA, USA). Molecular weights (kDa) are shown. (**B**) X-ray diffraction patterns of the lyophilized Omp fibrils. Shown are reflections in angstroms (Å). (**C**) CD spectra in the far UV region of Omp aggregates. Decoding for the used colors is given on the panel. (**D**) Deconvolution of CD spectra of the Omp aggregates. The content of α-helixes (α), β-sheets (β), β-turns (turn), and disordered (disord) structures is shown. Designations “E” and “S” in the names of proteins correspond to the orthologs from *E. coli* and *S. enterica*, respectively.

One of the typical properties of the amyloids is their resistance to ionic detergents and proteases [42,43]. We analyzed the effects of cold and hot 2% sodium dodecyl-sulfate (SDS) and trypsin protease on the in-vitro-obtained fibrillar aggregates of the *E. coli* and *S. enterica* OmpC and OmpF (Figure 3A). Samples containing fibrils of the corresponding four proteins were mixed with sample buffer containing SDS (final concentration 2%) and either left at room temperature or boiled for 5 min. In parallel, the same four samples were studied for protease resistance using trypsin (see “Section 4”). After that, all processed samples were loaded onto the SDS-PAGE (sodium dodecyl sulfate—polyacrylamide gel electrophoresis) gel, and signals of proteins were detected using the Stain-Free™ technology (Bio-Rad, Hercules, CA, USA). In all cases, one can note an increase in the monomer fraction after boiling (Figure 3A: lanes with index “B”) in comparison with unboiled samples (lanes “U”), which indicates solubilizing the fibrillar fraction to a monomeric state. It is also seen that trypsin treatment does not cause complete digestion of any of the four analyzed proteins, though significant amounts of proteins are degraded if we compare the lanes obtained after the trypsin treatment with the corresponding lanes “B” (untreated boiled samples) (Figure 3A). The recombinant RopB protein that was not subjected to fibrillogenesis was used as a control and underwent complete trypsin digestion under similar conditions (Figure S2). Thus, all four studied Omps form detergent- and protease-resistant aggregates in vitro.

### 2.4. OmpC and OmpF of E. coli and S. enterica Form Bona Fide Amyloids In Vitro

To analyze the structural features of the studied fibrillar aggregates of Omps, the samples were also subjected to X-ray diffraction analysis (Figure 3B). In all four tested samples, we observed a typical reflection for *bona fide* amyloid fibrils: the 4.7 to 4.9 Å reflection that is assumed to arise [44] from the periodicity of the hydrogen-bonded β-strands oriented near perpendicular to the axis of the fibril, and the diffraction in the region of approximately 8 to 11 Å that is presumed to relate to the stacking of these sheets parallel to the axis of the fibril (Figure 3B). The weaker ring at ≈3.7 Å resolution may be the higher-order reflection along the fibril axis, as described in the work [45]. It is also known that the reflection of 3.7 Å may correspond to paraffin [46] that is used to attach the sample.

To analyze the content of various elements of the secondary structure in the samples, we used the method of circular dichroism (CD). CD spectra recorded in the far UV region have a single minimum in the range of 216–230 nm for all tested samples, which is characteristic of proteins rich in β-structure forming the fibril backbone (Figure 3C). At the same time, it turned out that the CD spectra have different shapes and positions for different samples. We estimated the content of the different types of secondary structure in the samples using the CDPro software (https://sites.google.com/view/sreerama (accessed on 1 August 2023)) and the BeStSel webserver [47,48]. The results obtained confirmed the assumptions about the high content of β-sheets in the samples (from 36 to 43%). The smallest number of β-structures (36%), as well as the highest proportion of β-turns (25%) and disordered structures (35%), was observed for OmpC amyloids from *E. coli*. It can be assumed that it is regions outside the fibrillar backbone that are responsible for the interaction of these amyloids with each other and ensure their high tendency to cluster (Figure 1D).

Taken together, a wide array of methods used in our study, including morphological and structural analyses, detergent and protease treatment, and staining with CR and ThT dyes, demonstrated that in-vitro-obtained fibrillar aggregates of OmpC and OmpF from *E. coli* and *S. enterica* satisfy all criteria of *bona fide* amyloids.

### 2.5. OmpC and OmpF Amyloid Aggregates Are Toxic to Mammalian Cells

**Figure 4:**
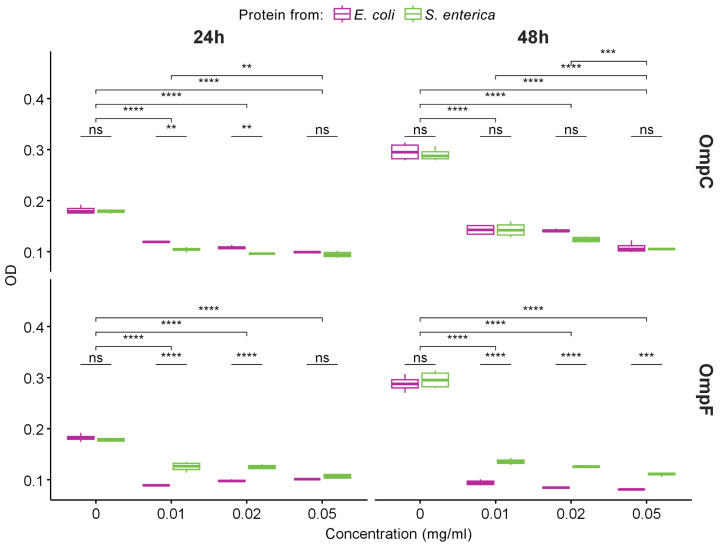
The data of MTT assay for evaluation of the metabolic activity of THP-1 cell lines exposed to different concentrations of fibrils obtained from OmpC (top row) and OmpF (bottom row) proteins of *E. coli* and *S. enterica* for 24 and 48 h. Color denotes bacterial species. Data are given as the mean ± SEM for four replicates. ** *p* ≤ 0.01, *** *p* ≤ 0.001, **** *p* ≤ 0.0001, ns—non-significant.

Since we demonstrated that OmpC and OmpF fibrils represent *bona fide* amyloids, we decided to analyze their toxicity to mammalian cells. Various amyloids are known to show toxicity to eukaryotic cells, which occurs mainly due to the ability of amyloid fibrils to bind cell membranes, leading to their disruption and cell death [49]. We tested the toxicity of fibrils formed by OmpC and OmpF of *E. coli* and *S. enterica* in vitro against human monocytic leukemia THP-1 cells (ATCC TIB-202). We used different concentrations of fibrils and two times of incubation: 24 and 48 h. All experiments were quadruplicated. The analysis of cells’ viability performed using the MTT assay (see “Section 4”) demonstrated that all four variants of fibrils caused statistically significant toxicity effects (Figure 4). Notably, *E. coli* and *S. enterica* OmpC fibrils showed the same toxicity, whereas the fibrils of the *E. coli* OmpF were more toxic than the fibrils formed by its ortholog from *S. enterica* (Figure 4). However, *E. coli* OmpC fibrils are less toxic than *E. coli* OmpF fibrils; this effect is cumulative and is better seen within 48 h. For *S. enterica* Omps, OmpC fibrils are more toxic than OmpF fibrils at first, but they level off after 48 h (Figure S3).

Thus, amyloid fibrils from four different Omps analyzed in our study exhibit toxicity to mammalian cells. This effect could potentially contribute to pathogenic properties of the corresponding bacterial species during their interaction with a eukaryotic host if OmpC and OmpF are able to adopt amyloid state in vivo.

### 2.6. OmpC and OmpF from E. coli and S. enterica Form Amyloid Fibrils Being Heterologously Expressed in the C-DAG System

**Figure 5:**
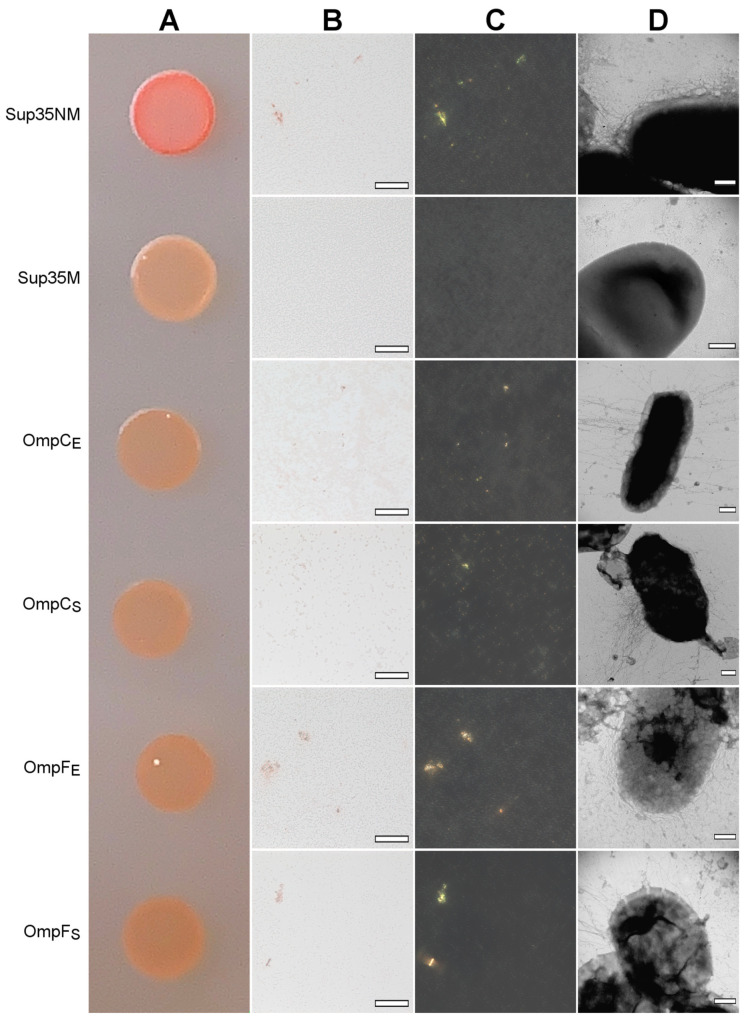
The heterologous secretion of the OmpC and OmpF proteins in the C-DAG system leads to amyloid formation. (**A**) CR plate with *E. coli* VS39 cells secreting OmpC and OmpF of *E. coli* and *S. enterica* to the cell surface. The cells secreting either Sup35NM (amyloid) or Sup35M (soluble) proteins were used as the positive and negative controls, respectively. (**B**,**C**) *E. coli* VS39 cells secreting OmpC and OmpF of *E. coli* and *S. enterica* form deposits that bind CR (**B**) and exhibit birefringence in polarized light (**C**). The scale bars are equal to 50 μm. (**D**) TEM images of the *E. coli* VS39 cells secreting OmpC and OmpF of *E. coli* and *S. enterica*. The scale bars are equal to 200 nm. Designations “E” and “S” in the names of proteins correspond to the orthologs from *E. coli* and *S. enterica*, respectively.

We found that all four analyzed Omps form *bona fide* amyloids in vitro. Next, we tested whether these proteins form amyloids that are heterologously secreted to the surface of *E. coli* cells using the C-DAG (Curli-Dependent Amyloid Generator) system [50]. As a result of this experiment, we have found that overexpression of all analyzed proteins, OmpC and OmpF from *E. coli* and *S. enterica*, in the *E. coli* strain VS39 has the same phenotypic manifestation, resulting in the orange color of colonies on the plates with cultural media containing CR (see “Section 4”). Such a phenotype suggests that VS39 cells secreting proteins of interest bind CR (Figure 5A). Polarized light microscopy of colonies confirmed this observation. Indeed, VS39 colonies secreting Omps contained congophylic deposits (Figure 5B) that, in contrast to the negative control (VS39 cells secreting soluble Sup35M protein), exhibited birefringence under polarized light (Figure 5C). While both samples secreting OmpC proteins from *E. coli* and *S. enterica* mostly contained relatively small CR-positive deposits, the samples secreting OmpF proteins from *E. coli* and *S. enterica* contained larger deposits similar to the positive control (VS39 secreting Sup35NM amyloid) (Figure 5C). Examination of colonies by transmission electron microscopy (TEM) confirmed amyloid fibril formation by VS39 cells secreting all four Omps: these cells, in contrast to the negative control (cells secreting Sup35M), contained long, thin fibrils at their surfaces (Figure 5D).

Thus, OmpC and OmpF from *E. coli* and *S. enterica* adopt the amyloid state being secreted to the surface of VS39 *E. coli* cells in the C-DAG system. Nevertheless, C-DAG is a very informative but heterologous system for the secretion of a protein of interest to the cell surface, which is provided by its fusion with a specific bipartite N-terminal peptide governing protein-of-interest to the cell surface and co-overexpression with a specific nonameric channel, CsgG, providing translocation through the outer membrane [50]. Thus, in order to better understand whether native Omps are capable of aggregating in vivo, it is important to analyze the effects of overproduction of OmpC and OmpF without fusion with heterologous peptides changing their subcellular localization.

### 2.7. OmpC and OmpF Form Aggregates In Vivo

**Figure 6:**
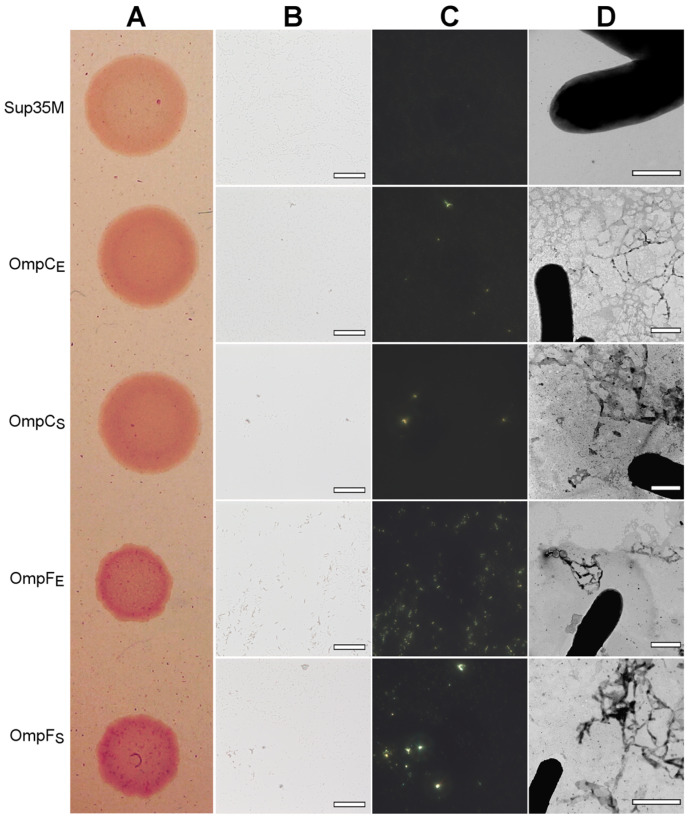
The effects of overproduction of the untagged, full-length OmpC and OmpF proteins with endogenous N-terminal signal peptides in the *E. coli* BL21 (DE3) strain. (**A**) CR plate with *E. coli* BL21 (DE3) colonies overproducing corresponding proteins. Strain overproducing soluble Sup35M protein was used as the negative control. (**B**,**C**) *E. coli* BL21 (DE3) cells overproducing OmpC and OmpF proteins of *E. coli* and *S. enterica* form deposits that bind CR (**B**) and exhibit birefringence in polarized light (**C**). The scale bars are equal to 50 μm. (**D**) TEM images of the *E. coli* BL21 (DE3) cells overproducing OmpC and OmpF proteins of *E. coli* and *S. enterica*. The scale bars are equal to 800 nm. In all experiments shown in the figure, cells were grown on YESCA plates for 48 h at 30 °C. Designations “E” and “S” in the names of proteins correspond to the orthologs from *E. coli* and *S. enterica*, respectively.

To perform overproduction of OmpC and OmpF in *E. coli* and *S. enterica*, we constructed a series of plasmids containing genes encoding full-length, untagged Omps with endogenous N-terminal signal peptides under the control of the IPTG (isopropyl β-D-1-thiogalactopyranoside)-inducible promoter (see “Section 4”). We overproduced *E. coli* and *S. enterica* OmpC and OmpF in the *E. coli* BL21 (DE3) strain and analyzed the effects of their overproduction. We found that overproduction of all four Omps causes a moderate congophylic phenotype (OmpF overproducing colonies exhibited a brighter color than those overproducing OmpC) on the inducible YESCA media supplemented with CR (Figure 6A), suggesting protein aggregate formation. Moreover, polarized light microscopy examination of the colonies revealed an increase in the formation of the congophylic birefringent deposits (Figure 6B,C), indicating the induction of amyloid formation by overproduction of all four proteins. Notably, while overproduction of OmpC in *E. coli* and *S. enterica* as well as OmpF in *S. enterica* led to the formation of few deposits, overproduction of OmpF from *E. coli* caused the formation of numerous birefringent dots (Figure 6C). TEM analysis of the samples did not demonstrate any increase in the fibril formation at the surface of *E. coli* cells for all analyzed Omps, indicating that the deposits comprising these proteins are located separately in the extracellular space (Figure 6D).

Thus, overproduction of the full-length OmpC and OmpF from *E. coli* and *S. enterica* with endogenous signal peptides increases CR binding by *E. coli* colonies and causes the formation of amyloid-like extracellular aggregates.

**Figure 7:**
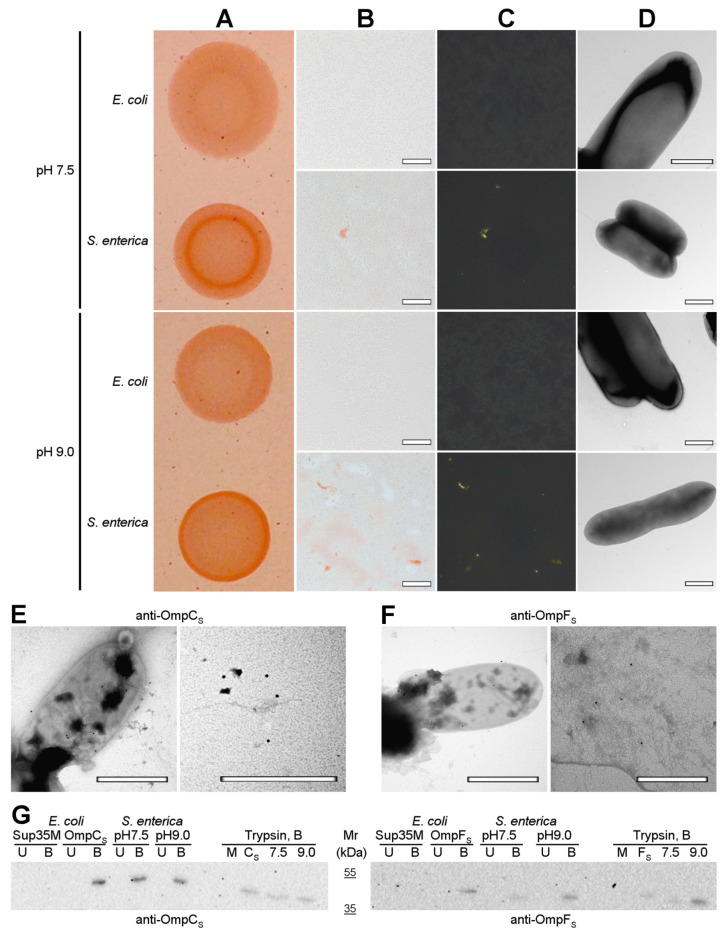
OmpC and OmpF aggregate in *Salmonella* cells under native conditions in vivo. (**A**) Shown are colonies of *E. coli* BL21 (DE3) and *S. enterica* strains grown on the YESCA plates supplemented with CR for 48 h at 30 °C at different pHs (7.5 and 9.0). (**B**,**C**) Transmitted and polarized light microscopy of the corresponding colony samples stained with CR. The scale bars are equal to 50 μm. (**D**) TEM images of *E. coli* BL21 (DE3) and *S. enterica* strains grown on the YESCA plates supplemented with CR for 48 h at 30 °C at different pHs (7.5 and 9.0). The scale bars are equal to 800 nm. (**E**,**F**) TEM images of the *S. enterica* cells and extracellular material labeled with either anti-*S. enterica* OmpC (**E**) or anti-*S. enterica* OmpF (**F**) antibodies visualized by gold-conjugated secondary antibodies. The scale bars are equal to 800 nm. (**G**) Western blot analysis of the aggregation of OmpC and OmpF in *S. enterica* cells grown at different pHs (7.5 and 9.0). Left—antibody against OmpC of *S. enterica* was used; right—antibody against OmpF of *S. enterica* was used. B—boiled samples, U—unboiled samples. Sup35M—negative control (*E. coli* cells overproducing Sup35M), OmpC_S_ and OmpF_S_—positive controls (*E. coli* cells overproducing either OmpC_S_ or OmpF_S_, respectively). pH7.5 and pH9.0—protein samples obtained from *S. enterica* cells grown at corresponding pHs. Trypsin, B—samples treated with trypsin and then boiled. M and C_S_/F_S_—controls (*E. coli* cells overproducing Sup35M and either OmpC_S_ or OmpF_S_). Molecular weights (kDa) are shown.

Next, we analyzed the formation of amyloid-like aggregates by OmpC and OmpF in vivo under native conditions. We compared the phenotypes of the *E. coli* BL21 (DE3) and *S. enterica* strains used in this study on the YESCA media supplemented with CR with different pHs: 7.5 and 9.0 (Figure 7A). We found that, in contrast to *E. coli* BL21 (DE3), *S. enterica* exhibits a congophylic phenotype, which is more pronounced at the plates with pH 9.0, where strong CR accumulation occurs by the periphery of the colonies (Figure 7A). Polarized light microscopy demonstrated that *S. enterica* cultures grown at both pH conditions contain amyloid-like birefringent deposits (Figure 7B,C). As in the case of overproduction of Omps in *E. coli* BL21 (DE3) cells (Figure 6), using TEM, we did not detect an increase in the fibril formation directly at the surface of *S. enterica* cells grown neither at pH 7.5 nor pH 9.0 (Figure 7D). Thus, *S. enterica* produces extracellular amyloid-like deposits under native conditions.

To localize OmpC and OmpF proteins, we performed immunoelectron microscopy (immuno-TEM) analysis. We found that both anti-*S. enterica* OmpC (Figure 7E) and anti-*S. enterica* OmpF (Figure 7F) antibodies exhibit two different variants of localization: they bind the surface of *S. enterica* cells corresponding to the outer membrane β-barrel state of OmpC and OmpF (Figure 7E,F. left images) and bind extracellular deposits located separately from cells (Figure 7E,F. right images), probably containing aggregated proteins that agree with the polarized light microscopy data (Figure 7B,C).

To detect whether the aggregated states of *S. enterica* OmpC and OmpF formed under native conditions in vivo possess other properties typical for amyloids, we analyzed their resistance to treatment with ionic detergents and proteases. We extracted protein lysates from *S. enterica* cultures grown at different pHs and treated them with cold or hot 2% SDS or trypsin followed by SDS-PAGE, transfer of proteins to the PVDF membrane, and western blot hybridization with antibodies specific to OmpC and OmpF of *S. enterica* (see “Section 4”). The results of the experiment demonstrated (Figure 7G) that both OmpC and OmpF obtained from *S. enterica* samples grown at different pHs are present in the detergent-resistant aggregate fraction under native conditions, which agrees with the data previously obtained in *E. coli* [28]. These aggregates are resistant to treatment with SDS at room temperature but solubilized by boiling with SDS (Figure 7G, lanes pH7.5 and pH9.0). Moreover, the aggregates containing OmpC and OmpF obtained from *S. enterica* samples grown at different pH exhibit resistance to trypsin treatment (Figure 7G, trypsin section, lanes 7.5 and 9.0). Notably, amounts of detergent- and protease-resistant aggregates of OmpF increase in the protein samples of *S. enterica* culture grown at pH9.0 in comparison with pH7.5, while OmpC aggregates do not demonstrate the same effect (Figure 7G, lanes pH7.5 and pH9.0; Trypsin section, lanes 7.5 and 9.0). Note that overproduction of *S. enterica* OmpC and OmpF in *E. coli* BL21 (DE3) strain also causes the formation of detergent- and protease-resistant aggregates by these proteins (Figure 7G, lanes OmpC_S_, OmpF_S_, and Trypsin section, lanes C_S_ and F_S_). Thus, OmpC and OmpF are able to adopt amyloid-like aggregated states both under overproduction and in native conditions in vivo.

## 3. Discussion

Despite the fact that Omps have been primarily known for a long time as the transmembrane pores with β-barrel structure providing diffusion of different molecules through the membrane [51,52], recent studies demonstrate that several of them are capable of forming supramolecular assemblies with amyloid or amyloid-like properties. For example, a number of outer membrane porins from bacterial species belonging to the order *Rhizobiales* were bioinformatically predicted to be potentially amyloidogenic [53]. Among *Rhizobiales* proteins, the RopA and RopB Omps of *Rhizobium leguminosarum* root nodule bacterium were experimentally proven to form *bona fide* amyloids in vitro and extracellular amyloid fibrils in vivo that are likely to modulate the interaction of Rhizobia with its multicellular host, *Pisum sativum* L. [33,54]. The *Mannheimia haemolytica* Omp2-like outer membrane protein was shown to participate in the formation of extracellular fibrils that bind CR and in the adhesion to the adenocarcinomic human alveolar basal epithelial cells [55].

Another large set of experimental data supporting the formation of amyloids by porins has been obtained using common porins of the *Enterobacterales* species. The common porins of *E. coli*, OmpA, OmpC, and OmpF, were found to be present in the fraction of protein assemblies resistant to treatment with ionic detergents [28]. The *E. coli* OmpA porin, which is one of the most abundant proteins in cells of Gram-negative bacteria [2], forms aggregates in vitro, demonstrating several traits of amyloids, including fibrillar morphology and binding ThT [23]. The *Yersinia pseudotuberculosis* OmpF porin is bioinformatically predicted to be moderately disordered and potentially amyloidogenic [56], and being overproduced at low temperatures in *E. coli* is present at inclusion bodies with high β-sheet content [57]. The OmpC common porin of *E. coli* was demonstrated to form fibrils in vitro, possessing a set of amyloid properties, including protease resistance, and birefringence, in polarized light, though the authors did not analyze whether these fibrils possess a cross-β structure specific for amyloids [24]. The results of our experiments presented in this work not only confirm cross-β structure for the *E. coli* OmpC fibrils (Figure 3), but for the first time show that three other outer membrane proteins, *S. enterica* OmpC, *E. coli* OmpF, and *S. enterica* OmpF, form fibrils in vitro that possess all criteria of amyloids: detergent and protease resistance, specific effects upon binding with CR and ThT, a typical X-ray diffraction pattern, and a high content of β-sheets (Figure 1, Figure 2 and Figure 3). Thus, OmpC and OmpF from two different bacterial genera form *bona fide* amyloids, suggesting that the amyloid properties of these two common porins are conservative.

Despite OmpC and OmpF proteins from *E. coli* and *S. enterica* demonstrating high similarity and identity of their primary structures (more than 50% in all pairwise comparisons, Table S1), the amyloid fibrils formed by them in vitro have differences in morphology and physicochemical properties. In particular, according to TEM data, OmpC amyloids from *S. enterica* form a network of intertwined fibrils, while OmpC amyloids from *E. coli* form the largest clots (Figure 1D,E), which is in good agreement with the differences in the recorded values of the samples’ turbidity (Figure 1B). It should be noted that it is for the latter fibrils that we observed noticeable differences in the secondary structure from others, which may determine their highest tendency to clustering and highest resistance to unboiled SDS (Figure 1C, Figure 2C, and Figure 3D). It is important to note that despite the fact that OmpC proteins are more similar to each other than those of OmpF (identity 78.1%, similarity 84.1% vs. identity 58.5%, similarity 73.7%, respectively), the number of potentially amyloidogenic regions in *S. enterica* Omps is greater than the same for *E. coli* Omps. For example, *E. coli* OmpC has 10 potentially amyloidogenic segments, while *S. enterica* OmpC has 14. The OmpF proteins from *E. coli* and *S. enterica* contain 10 and 12 potentially amyloidogenic segments, respectively (Table S2). Though such an increase in the content of potentially amyloidogenic regions in *S. enterica* Omps is interesting, it does not show a clear relationship with the toxicity of the fibrils of these proteins to mammalian cells (Figure 4 and Figure S3) or with the intensity of CR staining of the *E. coli* colonies overproducing corresponding proteins (Figure 6). In general, amyloids of OmpC and OmpF from *E. coli* and *S. enterica* formed in vitro and in vivo have different morphological and structural properties. This is in good agreement with the literature data [40,58] that even small differences in the amino acid sequence of proteins can be the cause of their amyloid polymorphism.

The examination of the OmpC and OmpF amyloid properties demonstrated that they form extracellular amyloid fibrils that are fused with heterologous bipartite signal peptide, providing target protein secretion in the C-DAG [50] system (Figure 5). What is more important is that these porins not only form detergent- and protease-resistant aggregates binding CR and exhibiting birefringence being overproduced in *E. coli* (Figure 6 and Figure 7E) but are even present in the extracellular detergent- and protease-resistant aggregates formed under native conditions by *S. enterica* (Figure 7). Though the functional significance of such aggregate formation in vivo (Figure 7) needs additional clarification, the toxicity of OmpC and OmpF amyloids to mammalian cells (Figure 4) suggests that amyloid aggregate formation by OmpC and OmpF could represent a novel mechanism mediating the pathogenesis of *Enterobacteriaceae* species. The OmpC and OmpF are known to be involved in the processes controlling virulence and antibiotic resistance [59,60]. The *ompC* and *ompF* deletions result in the attenuation of the bacterial virulence and colonization capacity [7,61], which might reflect not only the loss of OmpC and OmpF porin activity but also their ability to form amyloids. Taken together, amyloid formation by OmpC and OmpF represents the unique structural dualism of the outer membrane porins of Gram-negative bacteria and, more globally, β-barrel proteins.

Such a formation of amyloids by β-barrel proteins does not represent some “exception to the rule” but is widespread [62]. In particular, amyloids or amyloid-like aggregates are formed by the following β-barrel proteins in vitro and, in several cases, in vivo: (i) Omps of Gram-negative bacteria discussed above, including OmpC and OmpF of *E. coli* and *S. enterica*, as well as RopA and RopB of *R. leguminosarum*; (ii) EBNA1 and E2 DNA-binding proteins of viruses [63,64]; (iii) proteins with cold shock domain (CSD) [65]; vicilin, a garden pea seed storage globulin with cupin-1 domains [34]; (iv) GFP-like proteins [66]; (v) SOD1 superoxide dismutase [67]; (vi) newt fibroblast growth factor nFGF-1 [68]; (vii) bovine β-lactoglobulin [69,70]. Amyloid formation by some β-barrel proteins is disease-associated. For example, SOD1 conversion to the amyloid state may lead to the development of amyotrophic lateral sclerosis in humans [71,72]. Most bacterial amyloids are involved in supraorganismal interactions with corresponding multicellular hosts [8]. Such interactions can be either neutral or beneficial in the case of legume-rhizobia symbiosis or pathogenic in the case of a set of *Enterobacteriaceae* species. The amyloid formation by Omps, including OmpC and OmpF, is likely to be functional for pathogenic bacteria and has a detrimental effect on host cells, at least in model conditions. A question that currently remains unanswered is the folding pathways of β-barrel proteins to amyloids [62]. Although some β-barrel proteins form oligomeric assemblies [73,74,75,76,77] and β-barrel oligomers are identified as common intermediates preceding the formation of amyloid structures [78], it is still unclear whether amyloids are formed from folded β-barrels or whether these are two completely independent alternative folding pathways for the respective proteins containing β-barrel domains.

To conclude, we demonstrated that OmpC and OmpF from *E. coli* and *S. enterica* are capable of forming toxic aggregates in vitro that have all the properties of *bona fide* amyloids: fibrillar morphology, specific effects upon staining with CR and ThT probes, characteristic X-ray diffraction pattern, high content of β-sheets, and resistance to treatment with ionic detergents and proteases. We showed that these four Omps exhibit amyloid properties that are heterologously overexpressed in the C-DAG system. We found that overproduction in *E. coli* cells of OmpC and OmpF from *E. coli* and *S. enterica* increases CR binding by colonies and causes the formation of congophylic birefringent deposits. The OmpC and OmpF proteins from *S. enterica* form aggregates resistant to treatment with SDS and trypsin in *E. coli* cells under overexpression conditions, which agrees with increasing CR binding and birefringence by *E. coli* cells. Moreover, OmpC and OmpF are detected in the extracellular amyloid-like detergent and protease-resistant aggregates formed by *S. enterica* in vivo under native conditions.

## 4. Materials and Methods

### 4.1. Microbial Strains and Plasmids

The *E. coli* strains DH5α [79] and BL21 (DE3) (New England Biolabs, Ipswich, MA, USA) were used for plasmid amplification and protein production, respectively. The *E. coli* strain VS39 was used for protein secretion in the curli-dependent amyloid generator (C-DAG) system [50]. The *S. enterica* subsp. *enterica* var. Issatschenko strain 29/1 from the Russian Collection of Agricultural Microorganisms (RCAM) (http://62.152.67.70/cryobank/login.jsp (accessed on 24 July 2023)) was used [80].

To construct plasmids for the overproduction of target proteins, OmpC and OmpF from *E. coli* and *S. enterica*, fused with the C-terminal 6×His tag, corresponding genes without sequences encoding N-terminal signal peptides were PCR-amplified by using the matching pairs of primers (Table S3) and genomic DNA of the *E. coli* BL21 (DE3) and *S. enterica* subsp. *enterica* var. Issatschenko 29/1 strains, respectively.

To analyze the phenotypic effects of overproduction of target proteins, OmpC and OmpF from *E. coli* and *S. enterica*, corresponding full-length open reading frames, including sequences encoding N-terminal signal peptide and endogenous nonsense codons at the end of the open reading frame, were amplified by using the matching pairs of primers (Table S4) and genomic DNA of the *E. coli* BL21 (DE3) and *S. enterica* subsp. *enterica* var. Issatschenko 29/1 strains, respectively. For phenotypic analysis, *E. coli* BL21 (DE3) cells overproducing corresponding proteins were grown on the inducing YESCA [81] plates supplemented with CR for 48 h at 30 °C. To obtain a clearer phenotype of colonies grown on CR-containing plates, it is useful to additionally incubate plates for 48 h at 4 °C.

The cloning of the PCR-amplified fragments into the pLATE vector was performed according to the manufacturer’s protocol (Thermo Fisher Scientific, Waltham, MA, USA). The correctness of the plasmids obtained was verified by sequencing with primers provided by the manufacturer (Thermo Fisher Scientific, Waltham, MA, USA).

For the analysis of the amyloid properties of targeted proteins in the C-DAG system [50], corresponding plasmids were constructed on the basis of the pVS72 vector [50] using primer pairs (Table S5). The pVS72-based plasmids for secretion of the control Sup35NM (amyloid) and Sup35M (soluble) proteins were obtained previously [50,82].

### 4.2. Protein Production, Purification, and Fibrillogenesis

The production of recombinant OmpC and OmpF proteins from *E. coli* and *S. enterica* was carried out in *E. coli* strain BL21 (DE3) (New England Biolabs, Ipswich, MA, USA) grown in 2TY liquid media (16 g/L tryptone, 10 g/L yeast extract, 5 g/L NaCl). We used 0.1 mM isopropyl β-D-1-thiogalactopyranoside (IPTG, Thermo Fisher, Waltham, MA, USA) to induce overproduction of proteins. After the induction of overproduction, cultures were grown for 4 h at 37 °C. The 6×His-tagged proteins were purified in the presence of 8 M urea with the use of a Ni-NTA (nitrilotriacetic acid) agarose (Invitrogen, Carlsbad, CA, USA) column according to the protocol [83] without the Q Sepharose purification step. Purified proteins were concentrated with ethanol.

For the preparation of Omp fibrils, the proteins were dissolved in 50% 1,1,1,3,3,3-Hexafluoro-2-propanol (HFIP, Sigma-Aldrich, Saint-Louis, MO, USA) and incubated for 7 days at 37 °C as previously described [33,34]. Afterward, the HFIP was evaporated under a stream of nitrogen, and the samples were stirred for an additional 7 days.

### 4.3. Congo Red Staining and Polarized Light Microscopy

The saturated CR (Sigma, USA) solution filtered through 45 µm filter (Millipore, Burlington, MA, USA) was used for CR staining of samples. Stained samples on the microscopic slides were dried on air and rigorously washed with 70% ethanol. Zeiss Axio Imager A2 (Carl Zeiss, Oberkochen, Germany) polarized light microscope equipped with 40× dry objective and crosspolarizers was used.

### 4.4. Transmission Electron Microscopy and Immunodetection

The transmission electron microscope Libra 120 (Libra 120, Carl Zeiss, Oberkochen, Germany) was applied to visualize the studied aggregates. Samples were put on the copper grids coated with formvar/carbon films (Electron Microscopy Sciences, Hatfield, PA, USA) and stained by a 1% aqueous solution of uranyl acetate.

Immunoelectron microscopy examination (immuno-TEM) of *S. enterica* cells was performed according to the protocol [84,85] with modifications [33]. A Jeol JEM-1400 transmission electron microscope (JEOL Corp., Tokyo, Japan) equipped with a Veleta CCD camera (Olympus-SIS, Münster, Germany) was used. Rabbit anti-*S. enterica* OmpC or anti-*S. enterica* OmpF (PrimeBioMed LLC, Moscow, Russia) primary antibodies were used, and a secondary antibody conjugated with gold particles (goat anti-rabbit immunoglobulin G (IgG)–gold (Electron Microscopy Sciences, Hatfield, PA, USA)) was applied. Grids (Figure S4A,B) incubated with an empty medium (but with the primary antibody and with gold-conjugated secondary antibody) and grids (Figure S4C,D) incubated with *S. enterica* cells and extracellular material labeled only with gold-conjugated secondary antibody were used as negative controls.

### 4.5. Confocal Laser Scanning Microscopy

Confocal laser scanning microscope Olympus FV 3000 (Olympus, Tokyo, Japan) and oil immersion objective with a 60× magnification, the numerical aperture NA 1.42, and laser with excitation line 405 nm were applied to confirm the staining of Omp aggregates with ThT.

### 4.6. Preparation of the Samples of Omp Amyloids with ThT

Thioflavin T (ThT) UltraPure Grade (AnaSpec, Fremont, CA, USA) without after-purification was used. ThT-fibril-tested solutions were prepared by equilibrium microdialysis using a Harvard Apparatus/Amika device (Harvard Apparatus, Holliston, MA, USA). Equilibrium microdialysis was performed with a concentration of aggregates of about 0.5 mg/mL and an initial concentration of ThT of about 32 μM. A spectroscopic study of the sample and reference solutions prepared by the proposed approach allowed us to determine the spectral and photophysical characteristics of ThT bound to tested amyloids [38].

### 4.7. Spectral Measurements

A U-3900H spectrophotometer (Hitachi, Tokyo, Japan) was applied to collect the absorption spectra of the samples. The absorption spectra of all samples and mixtures of samples with ThT were corrected by light scattering according to the standard procedure [86]. The turbidity of the samples containing fibrils was monitored by measuring absorbance at 530 nm.

The fluorescence spectra of the samples were measured using a Cary Eclipse spectrofluorimeter (Varian, Palo Alto, CA, USA). The fluorescence of ThT was excited at a wavelength of 440 nm and recorded at a wavelength of 480 nm. The recorded values of fluorescence intensity were corrected for the primary inner filter effect [87]. For Rayleigh light scattering (RLS) determination, the samples with fibrils were excited at 530 nm and registered at 530 nm.

Far-UV CD spectra (190–260 nm) were measured by a J-810 spectropolarimeter (Jasco, Tokyo, Japan) using a 1 mm path length cell. The secondary structure content of Omp samples was estimated by the C;DPro software (https://sites.google.com/view/sreerama (accessed on 1 August 2023)) and the BeStSel webserver [47,48].

For the calculation of the fluorescence lifetime of ThT bound to fibrils, the fluorescence decay curves were recorded by the spectrometer FluoTime 300 (PicoQuant, Berlin, Germany) with the Laser Diode Head LDH-C-440 (λ*_ex_* = 440 nm). The fluorescence of ThT was registered at λ*_em_* = 490 nm. The measured emission decays were fit to a multiexponential function using the standard convolute-and-compare nonlinear least-squares procedure [88]. In this method, the convolution of the model exponential function with the instrument response function (IRF) was compared to the experimental data until a satisfactory fit was obtained. The fitting routine was based on the nonlinear least-squares method. Minimization was performed according to Marquardt [89].

The photophysical characteristics of various types of Omp amyloid fibrils and ThT bound to these aggregates were determined based on the results of at least three independent experiments. The standard error of the mean was determined for a confidence interval of 0.95.

### 4.8. X-ray Diffraction Analysis

Droplets of Omps samples were placed between the ends of wax-coated glass capillaries (approximately 1 mm in diameter) separated at a distance of 1.5 mm. Fibril diffraction images of OmpC and OmpF from *E. coli* and *S. enterica* protein aggregates were collected on a XtaLab Synergy S (Rigaku, Tokyo, Japan) instrument with a HyPix detector and a PhotonJet microfocus X-ray tube using Cu Kα (1.54184 Å) radiation. The images were prepared using the CrysAlisPro (Agilent Technologies, Inc., Oxford, UK) data reduction package. The experiments were carried out at a 2° phi rotation, and the exposure time was 60 s.

### 4.9. Analysis of Detergent and Protease Resistance of Protein Aggregates

SDS–PAGE was performed according to the standard protocol [90]. Before loading on the SDS–PAGE gel, Laemli SDS–PAGE sample buffer containing SDS (final concentration 2%, Bio-Rad, Hercules, CA, USA) was added, and the samples were incubated at room temperature or boiled (as indicated in the text) for 5 min. For the experiments with trypsin digestion, the samples of the in-vitro-obtained OmpC and OmpF proteins from *E. coli* and *S. enterica* (1 mg/mL) were treated with trypsin (Sigma-Aldrich, Saint-Louis, MO, USA) at 1:60 to the total protein mass ratio for 20 min at 37 °C. The in-vitro-obtained RopB (21.6 kDa) protein sample that was not subjected to fibrillogenesis was used as the negative control. Gels with recombinant protein samples were stained using Stain-Free™ technology (Bio-Rad, Hercules, CA, USA).

In the experiments with detection of the aggregation of OmpC and OmpF *S. enterica* proteins in vivo, western blot analysis was carried out. The *S. enterica* cell culture was centrifuged (5000 rcf for 5 min), sample buffer with 2% SDS (final concentration) was added to the pellet, and sonication was performed (10 s, 40% power of Q125 Sonicator, no pulsation (QSonica, Newtown, CT, USA)). Samples exposed to trypsin were first subjected to trypsinolysis (as above), and then lysis with detergents and ultrasound treatment were performed. For wet transfer of SDS-PAGE gel onto the PVDF membrane (Amersham, Buckinghamshire, UK), the Mini Trans-Blot Cell system (Bio-Rad, Hercules, CA, USA) was used. To detect OmpC and OmpF *S. enterica* proteins, rabbit anti-*S. enterica* OmpC and anti-*S. enterica* OmpF (PrimeBioMed LLC, Moscow, Russia) antibodies, respectively, and secondary goat anti-rabbit IgG (H + L) antibodies (Thermo Scientific, Waltham, MA, USA) were used. The dilution of the primary antibody was 1:15,000; of the secondary antibody, it was 1:20,000. The ECL Prime Western Blotting Detection reagent (GE Healthcare, Chicago, IL, USA) and Bio-Rad ChemiDoc™ hardware and Image Lab Version 6.1 software (Bio-Rad, Hercules, CA, USA) were used to visualize signals of proteins.

### 4.10. C-DAG Assay

The analysis of the amyloid properties of OmpC and OmpF from *E. coli* and *S. enterica* in the curli-dependent amyloid generator (C-DAG) system was performed as described earlier [50]. To export proteins on the cell surface, *E. coli* strain VS39 [50] was transformed with constructed pExport(pVS72)-based plasmids, encoding OmpC and OmpF proteins from *E. coli* and *S. enterica* fused with the N-terminal CsgA signal sequence. The secretion of amyloid Sup35NM and soluble Sup35M proteins was used as a positive and a negative control of amyloid formation, respectively. The analysis of birefringence was performed using a Zeiss Axio Imager A2 (Carl Zeiss, Oberkochen, Germany) transmitted light microscope equipped with 40× dry objective and crosspolarizers after four days of incubation of colonies on CR-containing inducing plates at 30 °C. TEM was performed using a Libra 120, (Carl Zeiss, Oberkochen, Germany) miscroscope and samples obtained on inducing plates without CR dye.

### 4.11. Protein Toxicity Assay

The toxicity of the fibrils against a monocytic leukemia cell line THP-1 (ATCC TIB-202) was assessed as follows: The cells were plated in 96-well culture plates in 100 μL RPMI medium supplemented with 10% FBS, 50 μg/mL gentamycin, 0.05 mM β-mercaptoethanol, and 10% fibril buffer at a density of 5 × 105 cells/mL. A buffer without fibrils was used as a control. The final concentration of fibrils was 0.01 mg/mL. Cells were incubated at 37 °C and 5% CO_2_ for 24 h or 48 h. Then, the cell viability was tested according to the following protocol [91]. Briefly, 10 μL of MTT solution in PBS (5 mg/mL) were added to each well, and the plate was incubated at 37 °C and 5% CO_2_. After 4 h, 100 μL of SDS-HCl solution (10% SDS, 0.01N HCl) was added and incubated for another 18 h. The optical density at 570 nm was measured and subtracted from the optical density at 620 nm.

The experiments were performed in four replicates. Multiple group comparisons were processed using the one-way analysis of variance (ANOVA) method with the emmeans post hoc test (emmeans R package, https://github.com/rvlenth/emmeans (accessed on 10 August 2023)). The differences were considered significant at *p* < 0.05.

### 4.12. Bioinformatic Analysis of Protein Structure 

The Omps’ structures have been predicted using the AlphaFold (version 2.3.2) program [92]. Amyloidogenic regions of the proteins have been predicted with AmylPred2 [31]. An open-source version of Pymol version 2.5.0 has been used for visualization [93] (https://pymol.org/2/ (accessed on 1 August 2023)).

## Data Availability

All relevant data are within the paper and supplementary materials.

## Supplementary Materials

The following supporting information can be downloaded at: https://www.mdpi.com/article/10.3390/ijms242115522/s1.
