# Supplementary material for: OmpC and OmpF Outer Membrane Proteins of Escherichia coli and Salmonella enterica Form Bona Fide Amyloids
Source: Int J Mol Sci. 2023 Oct 24;24(21):15522. doi: 10.3390/ijms242115522 (PMC10649029; doi:10.3390/ijms242115522)
Supplement: Supplementary file 1 [file ijms-24-15522-s001.zip › ijms-2624393-supplementary.pdf]

International Journal of *Molecular Sciences*

MDPI

Supplementary Materials

OmpC and OmpF Outer Membrane Proteins of *Escherichia coli* and *Salmonella enterica* Form *Bona Fide* Amyloids

Mikhail V. Belousov[1,2], Anastasiia O. Kosolapova[1,2,+], Haidar Fayoud[1,2], Maksim I. Sulatsky[3], Anna I. Sulatskaya[3], Maria N. Romanenko[1], Alexander G. Bobylev[4], Kirill S. Antonets[1,2], Anton A. Nizhnikov[1,2,*]

1 All-Russia Research Institute for Agricultural Microbiology, 196608 St. Petersburg, Russia; m.belousov@arriam.ru (M.V.B.); kosolapova97@mail.ru (A.O.K.); haidar.fayoud@gmail.com (H.F.); m.romanenko@arriam.ru (M.N.R.); k.antonets@arriam.ru (K.S.A.)
2 Faculty of Biology, St. Petersburg State University, 199034 St. Petersburg, Russia
3 Institute of Cytology, Russian Academy of Sciences, 194064 St. Petersburg, Russia; m_sulatsky@mail.ru (M.I.S.); ansul@mail.ru (A.I.S.)
4 Institute of Theoretical and Experimental Biophysics, Russian Academy of Sciences, 142290 Pushchino, Russia; bobylev1982@gmail.com
* Correspondence: a.nizhnikov@arriam.ru or a.nizhnikov@spbu.ru
† Current address: MACE Laboratory, Environmental Engineering Institute, School of Architecture, Civil and Environmental Engineering, Swiss Federal Institute of Technology in Lausanne, 1015 Lausanne, Switzerland.

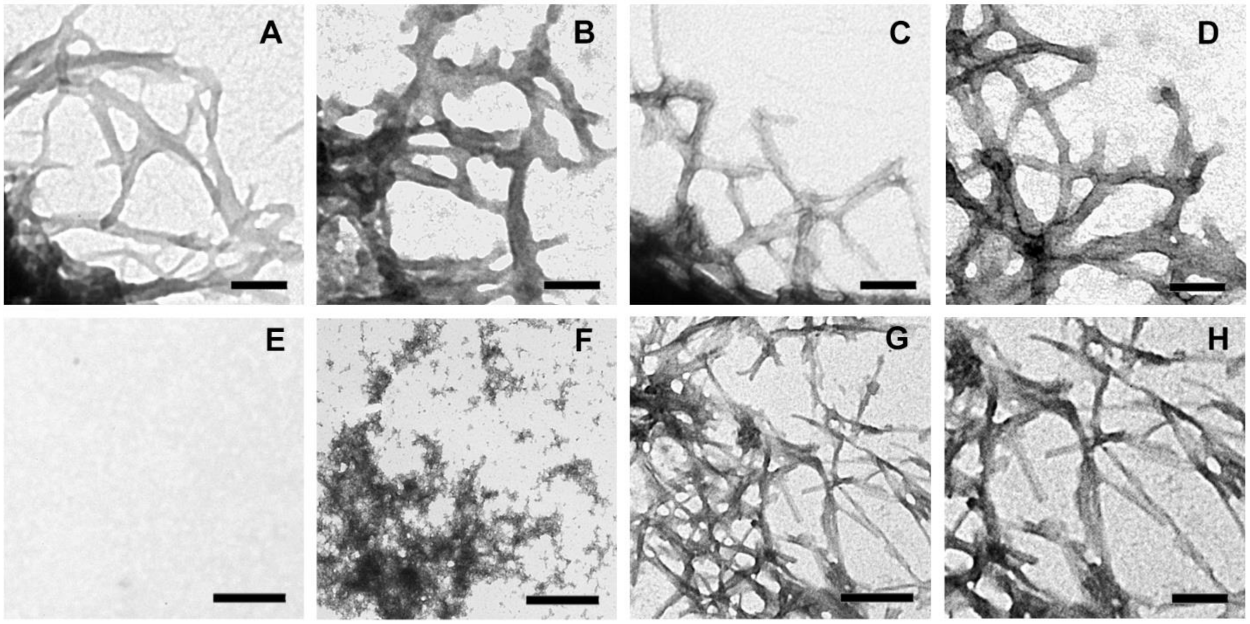

Figure S1. Visualization of the morphology of aggregates formed by Omps and lysozyme obtained *in vitro.* (**A-D**) TEM images of the fibrillar aggregates formed from (**A, B**) OmpC and (**C, D**) OmpF of (**A, C**) *E. coli* and (**B, D**) *S. enterica*, respectively. The scale bars at (**A-D**) are equal to 100 nm. (**E-H**) TEM images of the (**E**) soluble lysozyme, (**F**) lysozyme amorphous aggregates and (**G, H**) lysozyme amyloid fibrils. The scale bars of (**E-G**) are equal to 200 nm. The scale bar of the zoomed image at (**H**) is equal to 100 nm.

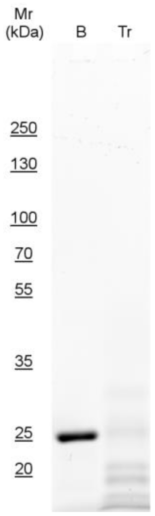

Figure S2. Analysis of the protease resistance of the recombinant RopB protein obtained *in vitro*. B – samples boiled with 2% SDS; Tr – treated with trypsin and boiled with 2% SDS. Gel stained using Stain-Free™ technology (Bio-Rad, Hercules, CA, USA). Corresponding molecular weights (kDA) are indicated.

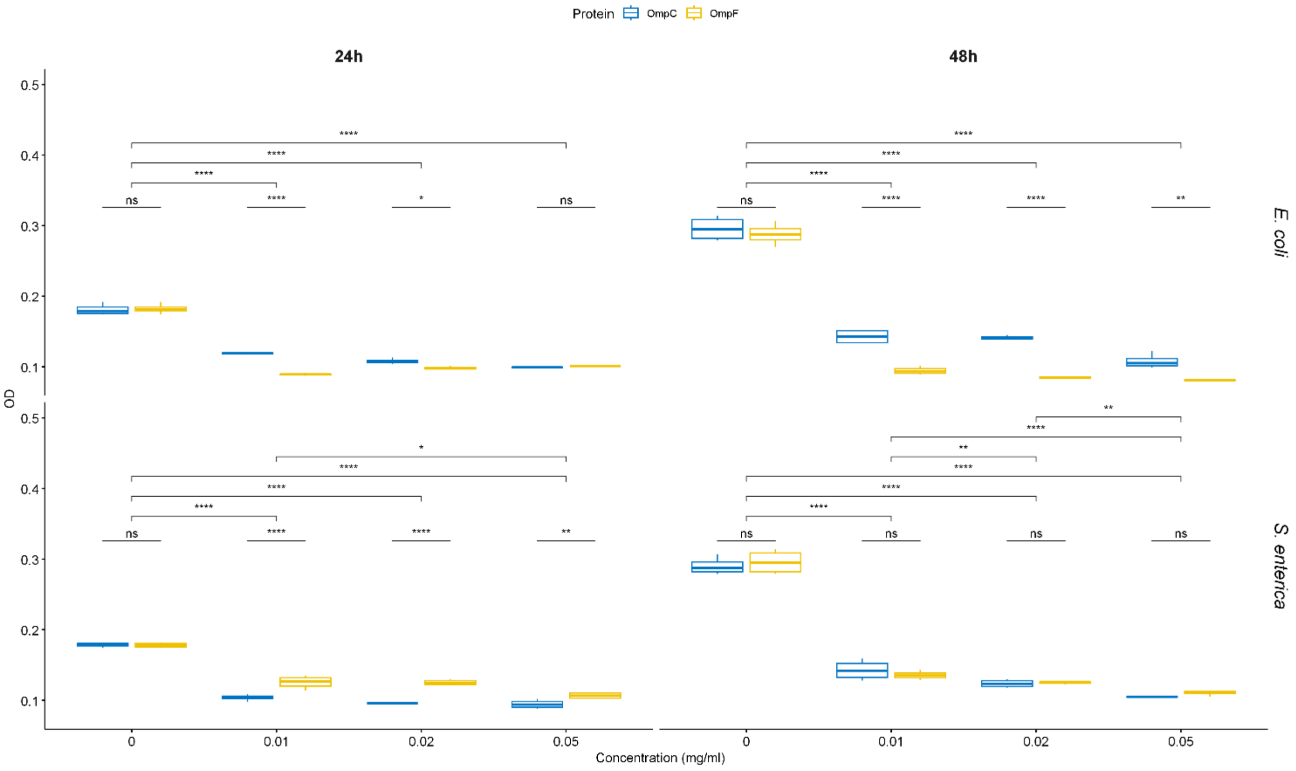

Figure S3. The data of MTT assay for the evaluation of the metabolic activity of THP-1 cell lines exposed to different concentrations of the fibrils obtained from OmpC and OmpF of *E. coli* (top row) and *S. enterica* (bottom row) for 24 and 48 hours. Color denotes the type of protein. Data are given as the mean ± SEM for four replicates. ** $p \leq 0.01$, *** $p \leq 0.001$, **** $p \leq 0.0001$, ns – non-significant.

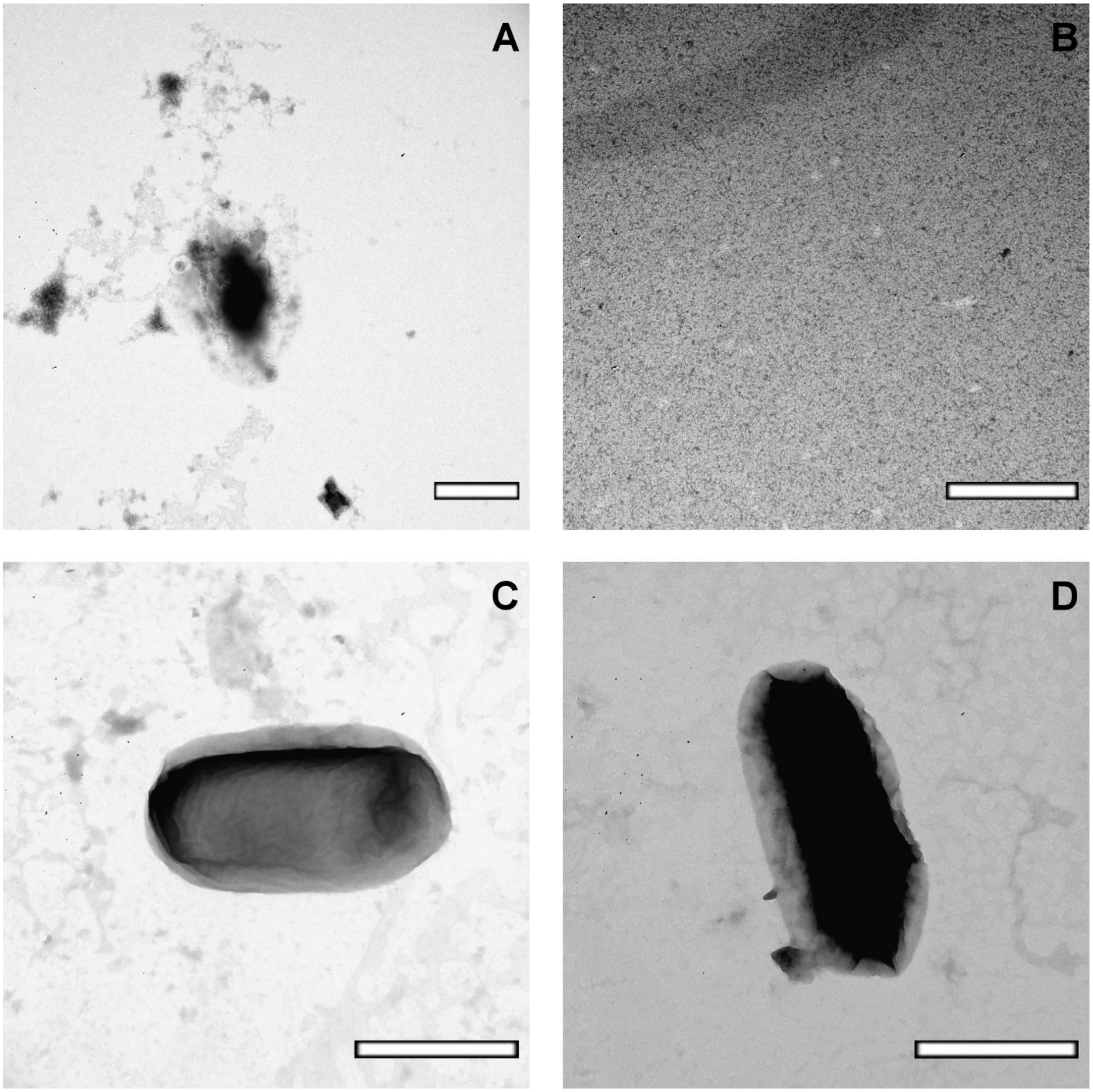

Figure S4. The negative controls for the immuno-TEM analyses shown at Figure 7E and F. (**A-B**) Grids incubated with an empty medium were treated with rabbit anti- *S. enterica* OmpC or anti- *S. enterica* OmpF (PrimeBioMed LLC, Moscow, Russia) primary antibody and a secondary antibody conjugated with gold particles (goat anti-rabbit immunoglobulin G (IgG)–gold (Electron Microscopy Sciences, Hatfield, PA, USA)) and visualized using TEM. The scale bars are equal to 800 nm. (**C-D**) TEM images of the *S. enterica* cells and extracellular material labeled only with gold-conjugated (goat anti-rabbit immunoglobulin G (IgG)–gold (Electron Microscopy Sciences, Hatfield, PA, USA)) secondary antibodies (without anti- *S. enterica* OmpC or anti- *S. enterica* OmpF primary antibodies). The scale bars are equal to 800 nm.

International Journal of *Molecular Sciences*

MDPI

Table S1. A comparison of several physicochemical properties of OmpC and OmpF from *E. coli* and *S. enterica.*

Protein	Species	Sequence	Number of amino acid residues	Mw (kDa)	pI	% Identity with				% Similarity with			
						OmpC$_E$	OmpC$_S$	OmpF$_E$	OmpF$_S$	OmpC$_E$	OmpC$_S$	OmpF$_E$	OmpF$_S$
OmpC	*E. coli*	AEVYNKDGNKLDLYGKVDGLHYFSDNKDVDGDQTYMRLGFKGETQVTDQLTGYGQWE<br>YQIQGNSAENENNSWTRVAFAGLKFQDVGSFDYGRNYGVVYDVTSWTDVLPEFGGDTYG<br>SDNFMQQRGNGFATYRNTDFFGLVDGLNFAVQYQGKNGNPSGEGFTSGVTNNGRDALR<br>QNGDGVGGSITYDYEGFGIGGAISSSKRTDAQNTAAYIGNGDRAETYTGGLKYDANNIYL<br>AAQYTQTYNATRVGSLGWANKAQNFEAVAQYQFDFGLRPSLAYLQSKGKNLGRGYDDE<br>DILKYVDVGATYYFNKNMSTYVDYKINLLDDNQFTRDAGINTDNIVALGLVYQFGHHHH<br>HH	353	39.187	4.598	-	289/370<br>(78.1%)	221/368<br>(60.1%)	195/364<br>(53.6%)	-	311/370<br>(84.1%)	265/368<br>(72.0%)	253/364<br>(69.5%)
OmpC	*S. enterica*	MAEIYNKDGNKLDLFGKVDGLHYFSDDKGSDGDQTYMRIGFKGETQVNDQLTGYGQWE<br>YQIQGNQTEGSNDSWTRVAFAGLKFADAGSFDYGRNYGVTYDVTSWTDVLPEFGGDTYG<br>ADNFMQQRGNGYATYRNTDFFGLVDGLDFALQYQGKNGSVSGENTNGRSLLNQNGDGY<br>GGSLTYAIGEGFSVGGAITTSKRTADQNNTADEHLYGNGDRATVYTGGLKYDANNIYLAA<br>QYSQTYNATRFGTSNGNNKSTSYGFANKAQNFEVVAQYQFDFGLRPSVAYLQSKGKDISN<br>GYGASYGDQDIVKYVDVGATYYFNKNMSTYVDYKINLLDKNDFTRDAGINTDDIVALGLV<br>YQFGHHHHHHG	366	40.345	4.656	-	-	215/373<br>(57.6%)	200/372<br>(53.8%)	-	-	258/373<br>(69.2%)	256/372<br>(68.8%)
OmpF	*E. coli*	AEIYNKDGNKVDLYGKAVGLHYFSKGNGENSYGGNGDMTYARLGFKGETQINSDLTGYG<br>QWEYNFQGNNSEGADAQTGNKTRLAFAGLKYADVGSFDYGRNYGVVYDALGYTDMLPE<br>FGGDTAYSDDFFVGRVGGVATYRNSNFFGLVDGLNFAVQYLGKNERDTARRSNGDGVGG<br>SISYEYEGFGIVGAYGAADRTNLQEAQPLGNGKKAEQWATGLKYDANNIYLAANYGETR<br>NATPITNKFTNTSGFANKTQDVLLVAQYQFDFGLRPSIAYTKSKAKDVEGIGDVDLVNYFE<br>VGATYYFNKNMSTYVDYIINQIDSDNKLGVGSDDTVAVGIVYQFGHHHHHH	347	37.964	4.825	-	-	-	207/354<br>(58.5%)	-	-	-	261/354<br>(73.7%)
OmpF	*S. enterica*	AEIYNKDGNKLDLYGKAVGRHVWTTTGDSKNADQTYAQIGFKGETQINTDLTGFGQWEY<br>RTKADRAEGEQQNSNLVRLAFAGLKYAEVGSIDYGRNYGIVYDVESYTDMAPYFSGETWG<br>GAYTDNYMTSRAGGLLTYRNSDFFGLVDGLSFGIQYQGKNQDNHSINSQNGDGVGYTMA<br>YEFDGFGVTAAYSNSKRTNDQQDRDGNGDRAESWAVGAKYDANNVYLAAVYAETRNM<br>SIVENTVTDTVEMANKTQNLEVVAQYQFDFGLRPAISYVQSKGKQLNGADGSADLAKYIQ<br>AGATYYFNKNMNVWVDYRFNLLDENDYSSSYVGTDDQAAVGITYQFGHHHHHH	348	38.765	4.627	-	-	-	-	-	-	-	-

Table S2. A comparison of the potentially amyloidogenic regions detected by the AmyPred2 method for OmpC and OmpF from *E. coli* and *S. enterica*.

Protein	Species	Potentially amyloidogenic regions identified by the AmyPred2 method*
OmpC	*E. coli*	19-24, 75-82, 94-103, 136-149, 194-196, 231-238, 265-266, 294-308, 313-314, 336-348
OmpC	*S. enterica*	20-20, 76-83, 95-104, 137-142, 145-150, 178-184, 187-192, 219-220, 230-236, 268-275, 306-320, 325-326, 329-330, 349-360
OmpF	*E. coli*	17-23, 85-87, 102-109, 128-138, 142-158, 185-192, 224-229, 257-265, 290-317, 331-342
OmpF	*S. enterica*	21-24, 75-82, 97-103, 141-147, 149-154, 176-180, 186-186, 222-230, 257-263, 297-304, 306-316, 334-341

*The start and end positions of amino acid residues in the region are indicated.

Table S3. The list of primers used in this work for the construction of plasmids based on pLATE vector for overproduction of C-terminally 6×His tagged proteins.

Plasmid	Primer name	Sequence	Target gene source	Target gene
pAc-OmpC_Ecoli*	FOmpCEcoliAl	AGAAGGAGATATAACTATGGCTGAAGTTTACAACAAAGACGG	*E. coli*	*ompC*
	ROmpCEcoliAl	GTGGTGGTGATGGTGATGGCCGAACTGGTAAACCAGACCCAG		
pAc-OmpC_Sal*	FOmpCSalAl	AGAAGGAGATATAACTATGGCTGAAATTTATAATAAAGACGGCAAC	*S. enterica*	*ompC*
	ROmpCSalAl	GTGGTGGTGATGGTGATGGCCGAACTGGTAAACCAGACCCAG		
pAc-OmpF_Ecoli*	FOmpFEcoliAl	AGAAGGAGATATAACTATGGCAGAAATCTATAACAAAGATGGCA	*E. coli*	*ompF*
	ROmpFEcoliAl	GTGGTGGTGATGGTGATGGCCGAACTGGTAAACGATACCCACA		
pAc-OmpF_Sal*	FOmpFSalAl	AGAAGGAGATATAACTATGGCAGAAATTTATAATAAAGATGGTAATAAG	*S. enterica*	*ompF*
	ROmpFSalAl	GTGGTGGTGATGGTGATGGCCGAACTGGTAAGTAATACCGACAG		

*These plasmids contain target genes (*ompC* or *ompF*) without sequence encoding N-terminal signal peptide but C-terminally fused with 6×His tag. The plasmids are used for the recombinant proteins production and purification.

Table S4. The list of primers used in this work for the construction of plasmids based on pLATE vector for overproduction of full-length untagged proteins.

Plasmid	Primer name	Sequence	Target gene source	Target gene
pAc-OmpC_sig_Ecoli*	FOmpCSigPAl	AGAAGGAGATATAACTATGAAAGTTAAAGTACTGTCCCTC	*E. coli*	*ompC*
	ROmpCSigPAl	GTGGTGGTGATGGTGATGGCCTTAGAACTGGTAAACCAGACCCA		
pAc-OmpC_sig_Sal*	FOmpCSigPAl	AGAAGGAGATATAACTATGAAAGTTAAAGTACTGTCCCTC	*S. enterica*	*ompC*
	ROmpCSigPAl	GTGGTGGTGATGGTGATGGCCTTAGAACTGGTAAACCAGACCCA		
pAc-OmpF_sig_Ecoli*	FOmpFSigPAl	AGAAGGAGATATAACTATGATGAAGCGCAATATTCTGGC	*E. coli*	*ompF*
	ROmpFSigPAl	GTGGTGGTGATGGTGATGGCCTTAGAACTGGTAAACGATACCCAC		
pAc-OmpF_sig_Sal*	FOmpFSalSigP	AGAAGGAGATATAACTATGATGAAGCGCAAAATCCTGGC	*S. enterica*	*ompF*
	ROmpFSalSigP	GTGGTGGTGATGGTGATGGCCTTAGAACTGGTAAGTAATACCGACA		

*These plasmids contain target genes (*ompC* or *ompF*) with sequence encoding its own N-terminal signal peptides and endogenous nonsense codon at the end of the open reading frame prior C-terminal 6×His tag containing in the pLATE vector.

Table S5. The list of primers used in this work for the construction of plasmids based on pExport (pVS72) vector for C-DAG system.

Plasmid	Primer name	Sequence	Target gene source	Target gene
pExport-OmpC_Ecoli	FOmpCEcoliCD	ACTAGCGGCCGCAGCTGAAGTTTACAACAAAGACGG	*E. coli*	*ompC*
	ROmpCEcoliCD	ACGTTCTAGATTAGAACTGGTAAACCAGACCCA		
pExport-OmpC_Salmonella	FOmpCSalCD	ACTAGCGGCCGCAGCTGAAATTTATAATAAAGACGGCAAC	*S. enterica*	*ompC*
	ROmpCSalCD	ACGTTCTAGATTAGAACTGGTAAACCAGACCCA		
pExport-OmpF_Ecoli	FOmpFEcoliCD	ACTAGCGGCCGCAGCAGAAATCTATAACAAAGATGGCA	*E. coli*	*ompF*
	ROmpFEcoliCD	ACGTTCTAGATTAGAACTGGTAAACGATACCCAC		
pExport-OmpF_Salmonella	FOmpFSalCD	ACTAGCGGCCGCAGCAGAAATTTATAATAAAGATGGTAATAAG	*S. enterica*	*ompF*
	ROmpFSalCD	ACGTTCTAGATTAGAACTGGTAAGTAATACCGACA		
